# The Effect and Safety of Iguratimod Combined With Methotrexate on Rheumatoid Arthritis: A Systematic Review and Meta-Analysis Based on a Randomized Controlled Trial

**DOI:** 10.3389/fphar.2021.780154

**Published:** 2022-01-18

**Authors:** Liuting Zeng, Ganpeng Yu, Kailin Yang, Wensa Hao, Hua Chen

**Affiliations:** ^1^ Department of Rheumatology and Clinical Immunology, Peking Union Medical College Hospital, Chinese Academy of Medical Sciences & Peking Union Medical College, National Clinical Research Center for Dermatologic and Immunologic Diseases (NCRC-DID), Key Laboratory of Rheumatology and Clinical Immunology, Ministry of Education, Beijing, China; ^2^ Department of Orthopedics, People’s Hospital of Ningxiang City, Ningxiang, China; ^3^ Beijing Anzhen Hospital, Capital Medical University, Beijing, China; ^4^ Institute of Material Medical: Chinese Academy of Medical Sciences & Peking Union Medical College Institute of Materia Medica, Beijing, China

**Keywords:** methotrexate, rheumatoid arthritis, systematic review, meta-analysis, iguratimod

## Abstract

**Background:** Rheumatoid arthritis (RA) is a chronic systemic autoimmune disease with inflammatory synovitis. Iguratimod (IGU) combined with methotrexate (MTX) therapy may have better efficacy and safety.

**Methods:** First, we searched randomized controlled trials (RCTs) of IGU + MTX in the treatment of RA through literature databases (such as PubMed, Corkland Library, CNKI, etc.) and then conducted RCT quality assessment and data extraction. Finally, we used RevMan 5.3 for meta-analysis, STATA 15.0 for publication bias assessment, and GRADE tool for the evidence quality assessment of primary outcomes. This systematic review and meta-analysis were registered in PROSPERO (CRD42021220780).

**Results:** This systematic review and meta-analysis included 31 RCTs involving 2,776 patients. Compared with MTX alone, the ACR20, ACR50, and ACR70 of IGU + MTX are higher, while DAS28 is lower [ACR20: (RR 1.55, 95% CI 1.14–2.13, *p* = 0.006); ACR50: (RR 2.04, 95% CI 1.57–2.65, *p* < 0.00001); ACR70: (RR 2.19, 95% CI 1.44–3.34, *p* = 0.00003); DAS28: (weighted mean difference (WMD) −1.65, 95% CI −2.39 to −0.91, *p* < 0.0001)]. Compared with MTX + leflunomide, IGU + MTX has no significant difference in improving ACR20, ACR50, ACR70, but IGU + MTX improves DAS28 more significantly [ACR20: (RR 1.09, 95% CI 0.79–1.89, *p* = 0.59); ACR50: (RR 1.07, 95% CI 0.64–1.78, *p* = 0.81); ACR70: (RR 1.17, 95% CI 0.44–3.10, *p* = 0.76); DAS28: (WMD −0.40, 95% CI −0.42 to −0.38, *p* < 0.0001)]. Compared with the MTX + tripterygium subgroup and MTX-only subgroup, the incidence of adverse events of the IGU + MTX group is of no statistical significance [MTX only: (RR 0.99, 95% CI 0.87–1.13, *p* = 0.90); MTX + Tripterygium: (RR 0.73, 95% CI 0.29–1.85, *p* = 0.50)]. However, compared with MTX + leflunomide, the incidence of adverse events in the IGU + MTX group was lower (RR 0.74, 95% CI 0.62–0.88, *p* = 0.0009). The quality of ACR70 was high; the quality of adverse events and ACR50 test was moderate.

**Conclusion:** Compared with conventional therapy, IGU + MTX may be a safer and more effective therapy for RA patients. When the intervention method is (IGU 25 mg Bid, MTX 10–25 mg once a week), and the intervention lasts for at least 12 weeks, the curative effect may be achieved without obvious adverse events.

## Introduction

Rheumatoid arthritis (RA) is a chronic systemic autoimmune disease with inflammatory synovitis, the cause of which is not yet clear, and it mainly damages the synovial tissue of the joints. It is characterized by the increase of interleukin (IL) and tumor necrosis factor (TNF), and the activation of T lymphocytes, which may lead to severe chronic inflammation of joints, and even erosion and destruction of cartilage, bone, and tendons (Scott et al., 2010; Wasserman 2011; Wasserman 2018). Epidemiological studies show that the incidence of RA worldwide is 0.5%–1% (Wasserman 2018; Otón and Carmona 2019). Although the incidence is not high, the number of patients is very large due to the long survival time of patients. In particular, many patients with RA have joint deformities in the late stage, causing paralysis, complete loss of labor, and occupation of a large number of medical and social resources (Hunter et al., 2017; Otón and Carmona 2019). The current goal of RA treatment is to alleviate patients’ clinical symptoms, reduce or prevent patients’ joint damage, and emphasize the early use of disease-modifying anti-rheumatic drugs (DMARDs), such as methotrexate and leflunomide (Burmester and Pope 2017; Ferro et al., 2017). In addition, some biological agents such as anti-TNF-α blockers, anti-IL antibodies, and CD20 monoclonal antibodies are also used in the treatment of RA (Burmester and Pope 2017; Liu et al., 2018).

In view of the complex pathogenesis of RA, a single drug often fails to effectively achieve the treatment goals. Therefore, major international and domestic guidelines recommend the use of combination therapy when a single DMARD treatment fails to meet the standard (Singh et al., 2016; Smolen et al., 2017; Luo et al., 2019). Combination therapy can improve the efficacy and reduce the incidence of adverse reactions, which is the main trend of RA treatment. The 2018 Chinese Rheumatoid Arthritis Diagnosis and Treatment Guidelines also mention that those who have not reached the standard after MTX single-agent standard treatment are recommended to be combined with another synthetic DMARD for treatment (Chinese Rheumatology Association, 2018). Iguratimod (IGU) was considered to be the preferred drug for combination therapy. A number of studies have confirmed that after the treatment of patients with poor efficacy of methotrexate (MTX) combined with IGU treatment, the efficacy indicators such as disease activity and bone metabolism have been significantly improved, and the incidence of adverse reactions is low (Ishiguro et al., 2013; Hara et al., 2014; Zhang et al., 2014; Ding et al., 2019; Suto et al., 2019). Recently, we conducted a multicenter, large-scale randomized controlled trial to investigate the efficacy and safety of the IGU combined with MTX group (group A) and the leflunomide combined with MTX group (group B) on the two groups at 12, 24, and 52 weeks after treatment and compared the ACR20 compliance rate, the improvement range of DAS28, and the incidence of adverse reactions after 52 weeks (Tian et al., 2020). The results show that IGU combined with MTX might be as effective as leflunomide combined with MTX in the treatment of patients with active RA, and the incidence of adverse events is low. At present, Chen et al. (2021) conducted a systematic review and meta-analysis of IGU + MTX treatment of RA, but only seven randomized controlled trials (RCTs) were included. A number of RCTs from other clinical research centers have also reported the effectiveness and safety of IGU combined with MTX in the treatment of RA (Lu 2014; Duan et al., 2015; Xia et al., 2016). However, the previous RCTs are often small-scale single-center clinical trials (Lu 2014; Duan et al., 2015; Shi et al., 2015; Xia et al., 2016). Therefore, this study would conduct a systematic review and meta-analysis of the effectiveness and safety of IGU combined with MTX in the treatment of RA for the first time, in order to provide solid evidence and new directions for clinical use and also provide new experience and direction for future RCTs.

## Materials and Methods

### Protocol

This meta-analysis were conducted strictly in accordance with the protocol registered in PROSPERO (CRD42021220780) and PRISMA guidelines (see Supplementary Materials).

### Literature Search Strategy

We searched Embase, PubMed, The ClinicalTrials.gov, VIP Database for Chinese Technical Periodicals, Wanfang Database on Academic Institutions in China, China National Knowledge Infrastructure (CNKI), Cochrane Library, China Biology Medicine (CBM), MEDLINE Complete, and Web of Science with the retrieval time up to December 2020. The search strategy is shown in Supplementary Table S1.

### Selection Criteria

#### Participants

The participants were patients with RA. The age, gender, and nationality of patients are not limited. The literature needs to mention clear diagnostic criteria for RA, with a balanced baseline and comparability.

#### Intervention

The treatment of the experimental group is IGU + MTX. The treatment of the control group was MTX alone or MTX combined with other therapies that did not contain IGU.

#### Outcomes

Primary outcomes are ACR20, ACR50, ACR70, 28 joint disease activity score (DAS28), and adverse events. Secondary outcomes are: (1) symptom-related outcomes: Health Assessment Questionnaire (HAQ), morning stiffness time (min), number of swollen joints, and number of tender joints; (2) bone protection-related outcomes: N-terminal osteocalcin (N-MID), total T-type procollagen amino terminal propeptide (T-PINP) levels, 25-hydroxy vitamin D [25(OH)D], and β-I collagen carboxy terminal peptide (β-CTX); (3) inflammation and immune response-related outcomes: rheumatoid factor (RF), erythrocyte sedimentation rate (ESR), C-reactive protein (CRP), anti-cyclic citrullinated peptides (CCP), and TNF-α; and (4) angiogenesis-related outcomes: VEGF.

#### Study Design

The study design was RCTs, with no limitations to publication time, language, quality, and publication status.

### Literature Screening and Data Extraction

In the first stage of literature screening, two reviewers independently conducted manual screening of the initially included clinical research literature. First, two reviewers read the title, abstract, and keywords and then exclude non-RCT, duplicate or identical papers, papers with obscure data, and papers whose full text cannot be obtained. In the second stage, after reading the full text of the literature, two reviewers further screened the literature that could eventually be included in the meta-analysis based on selection criteria. Finally, two reviewers independently extracted data from the literature. The differences between the two reviewers in the selection of literature and data extraction were resolved through consultation with the third reviewer.

### Risk of Bias Assessment

The “risk of bias” assessment tool recommended by the Cochrane Collaboration (Deeks et al., 2021a) is used to assess the risk of bias. The tools include 1) generation of random sequence; 2) allocation concealment; 3) blinding of subjects and intervention providers; 4) blinding of outcome evaluators; 5) incomplete outcome data; 6) selective outcome report; and 7) other sources of bias.

### Statistical Analysis

RevMan 5.3 software was used for risk-of-bias assessment and meta-analysis. First, the heterogeneity test was performed. The subgroup analysis was carried out based on the intervention measures of the control group. When the heterogeneity among the included studies was low (*p* > 0.1 and I^2^ ≤ 50%), the fixed-effect model was used; otherwise, the random-effect model was used (Deeks et al., 2021b). For continuous variables, when the measurement data units are different, the values differ greatly, or the measurement methods are different between different studies, the standardized mean difference (SMD) is used as the effect size indicator, and the other uses the mean difference (MD) as the effect size indicator. For dichotomous variables, the risk ratio (RR) is used as the effect size indicator. Each effect size is expressed with a 95% confidence interval (95% CI). The publication bias was detected by STATA 15 with the Egger method (continuous variable) and Harbord method (dichotomous variable) for primary outcomes. *p* > 0.1 is considered to have no publication bias. The GRADE tool was utilized to rate the quality of the evidence (GRADEpro 2015) according to the GRADE handbook (Schünemann et al., 2013).

## Results

### Results of the Search

**FIGURE 1:**
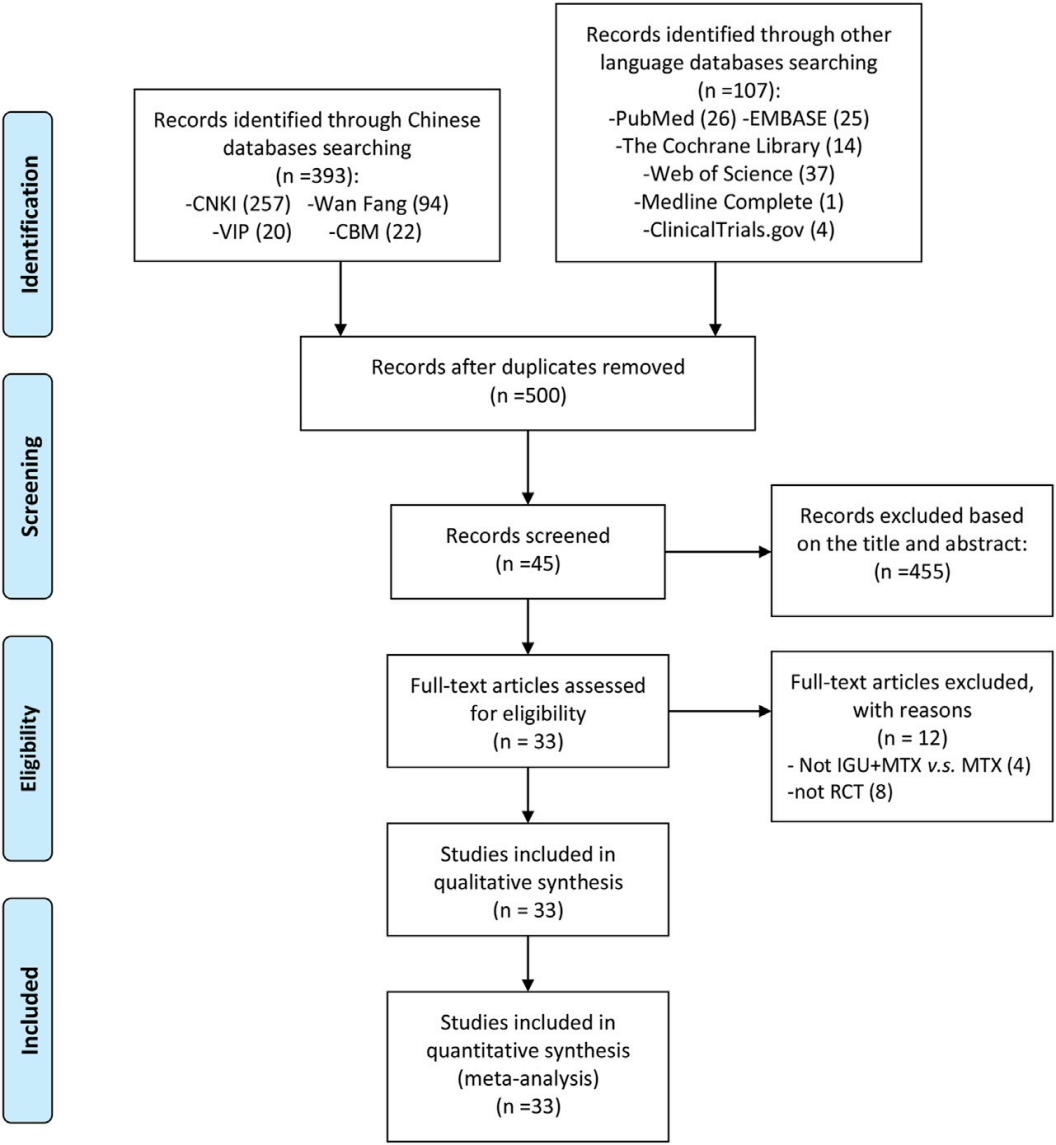
Flow diagram.

The total records identified through database searching and other sources were 500. According to the search strategy, a total of 45 articles were obtained through preliminary search. By eliminating duplicate documents, carefully reading the title and abstract, a total of 456 articles were excluded. After carefully reading the full text and comparing the selection criteria, 33 RCTs were screened out and finally included (Shi et al., 2015; Lu 2014; Duan et al., 2015; Xiong et al., 2020; Xu et al., 2015; Ding et al., 2019; Deng et al., 2017; Tian et al., 2020; Xie et al., 2018; Xu et al., 2017; Bi et al., 2019; Yan et al., 2018; Tian et al., 2017; Chen et al., 2018; Xiong et al., 2015; Mo et al., 2015; Fan et al., 2020; Zhao et al., 2018; Li et al., 2020; Li et al., 2019; Meng et al., 2017; Li et al., 2016; Xia et al., 2020; Meng et al., 2015; Mo et al., 2018; Meng et al., 2016; Wang et al., 2019; Qi et al., 2019; Ju et al., 2020; Zhao et al., 2016; Hara et al., 2014; Ishiguro et al., 2013; Xia et al., 2016) (Figure 1). The main reason why 12 studies were excluded is that eight studies are not RCTs (Yoshioka et al., 2016; Wang et al., 2017; Suto et al., 2019; Okamura et al., 2015a,b; Wang 2017; Meng et al., 2016; Gu et al., 2020), and the other four studies are not comparing IGU + MTX with MTX (Liu 2016; Xu et al., 2017; Lu and Liu 2018; Zhu 2019).

### Description of Included Trials

The study of Hara et al. (2014) (Ishiguro et al., 2013; Hara et al., 2014) was conducted in Japan. The participants in the other RCTs were from China. There are 10 RCTs with more than 100 participants (Hara et al., 2014; Xia et al., 2016; Meng et al., 2017; Tian et al., 2017; Chen et al., 2018; Li et al., 2019; Ju et al., 2020; Tian et al., 2020; Xia et al., 2020; Xiong et al., 2020); there are 20 RCTs ranging from 50 to 100 participants (Duan et al., 2015; Meng et al., 2015; Mo et al., 2015; Shi et al., 2015; Xu et al., 2015; Li et al., 2016; Meng et al., 2016; Zhao et al., 2016; Deng et al., 2017; Xu et al., 2017; Mo et al., 2018; Xie et al., 2018; Yan et al., 2018; Xiong et al., 2015; Zhao et al., 2018; Bi et al., 2019; Ding et al., 2019; Qi et al., 2019; Wang et al., 2019; Fan et al., 2020). The interventions in the control group were mostly MTX alone. The control group of Deng et al. (2017), Tian et al. (2020), Bi et al. (2019), and Meng et al. (2015) used MTX + leflunomide; the control group of Li et al. (2019), Xia et al. (2020), and Mo et al. (2018) used MTX + tripterygium; and the control group of Li et al. (2020) used MTX + adalimumab. Subgroup analysis would be based on the treatment of the control group. The details of study characteristics are presented in Table 1.

**TABLE 1:** The characteristics of the included studies.

Study	Sample size (female/male)		Intervention		Relevant outcomes	Mean age (years)		Disease duration (years)		Baseline CRP (mg/L)		Baseline ESR (mm/h)		Baseline DAS28		Duration
	Trial group	Control group	Trial group	Control group		Trial group	Control group	Trial group	Control group	Trial group	Control group	Trial group	Control group	Trial group	Control group	
Xiong et al. (2020)	51 (22/29)	51 (21/30)	IGU 25 mg Bid + MTX 10 mg once a week at the beginning; 12.5 mg twice a week after 2 weeks; 15 mg once a week after 4 weeks	MTX 10 mg once a week at the beginning; 12.5 mg twice a week after 2 weeks; 15 mg once a week after 4 weeks	Morning stiffness time, number of tender joints, number of swollen joints, N-MID, T-PINP, adverse events	48.21 ± 6.04	48.33 ± 5.93	1.98 ± 0.43	1.54 ± 0.39	-	-	-	-	-	-	24 weeks
Xu et al. (2015)	40 (23/17)	38 (24/14)	IGU 25 mg Bid + MTX 7.5–20 mg once a week	MTX 7.5–20 mg once a week	Morning stiffness time, number of tender joints, number of swollen joints, ESR, CRP, RF, adverse events	46.10 ± 17.09	43.28 ± 10.46	4.69 ± 0.58	4.34 ± 0.78	22.33 ± 5.17	24.55 ± 5.04	77.37 ± 18.26	74.05 ± 19.43	-	-	48 weeks
Ding et al. (2019)	38 (21/17)	38 (23/15)	IGU 25 mg Bid + MTX 10 mg once a week	MTX 10 mg once a week	β-CTX, 25(OH)D, N-MID, T-PINP	72.8 ± 2.7	72.6 ± 2.6	8.8 ± 2.8	8.6 ± 2.7	-	-	-	-	-	-	16 weeks
Deng et al. (2017)	90 (71/19)		IGU 25 mg Bid + MTX 10 mg once a week	MTX 10 mg once a week + Leflunomide 20 mg Qd	DAS28, HAQ, ESR, CRP, RF, CCP, adverse events	47.23 ± 15.62	4.75 ± 3.53			33 ± 15	34 ± 13	46 ± 13	45 ± 15	4.68 ± 0.07	4.62 ± 0.12	48 weeks
Tian et al. (2020)	107 (87/20)	100 (90/10)	IGU 25 mg Bid + MTX 10 mg once a week	MTX 10 mg once a week + Leflunomide 20 mg Qd	DAS28, HAQ, ESR, CRP, RF, CCP, adverse events	50 ± 10	49 ± 11	6.08 ± 6.25	6.75 ± 7.33	31 ± 30	39 ± 37	56 ± 27	59 ± 28	-	-	52 weeks
Xie et al. (2018)	39 (27/12)	39 (25/14)	IGU 25 mg Bid + MTX 10 mg once a week at the beginning; 12.5 mg twice a week after 2 weeks; 15 mg once a week after 4 weeks	MTX 10 mg once a week at the beginning; 12.5 mg twice a week after 2 weeks; 15 mg once a week after 4 weeks	DAS28, TNF-α, VEGF, adverse events	62.89 ± 4.57	62.74 ± 3.96	6.41 ± 2.16	7.35 ± 1.87	-	-	-	-	6.75 ± 1.69	6.84 ± 1.87	16 weeks
Xu et al. (2017)	42 (23/19)	41 (22/19)	IGU 25 mg Bid + MTX 7.5–20 mg once a week	MTX 7.5–20 mg once a week	DAS28, Morning stiffness time, ESR, CRP	46.34 ± 2.29	46.19 ± 2.57	4.72 ± 0.61	4.68 ± 0.59	57.37 ± 12.72	58.95 ± 12.16	69.95 ± 10.92	69.75 ± 10.91	6.92 ± 2.91	6.72 ± 2.94	48 weeks
Bi et al. (2019)	30 (24/6)	30 (24/6)	IGU 25 mg Bid + MTX 10 mg once a week	MTX 10 mg once a week + Leflunomide 20 mg Qd	DAS28, VEGF, adverse events	53.10 ± 12.90	54.60 ± 11.88	-	-	-	-	-	-	4.53 ± 0.71	4.43 ± 0.68	12 weeks
Yan et al. (2018)	35 (23/12)	35 (23/12)	IGU 25 mg Bid + MTX 10 mg once a week	MTX 10 mg once a week	TNF-α, β-CTX, T-PINP, N-MID, 25(OH)D, adverse events	56 ± 7	56 ± 7	11.6 ± 2.7	11.2 ± 2.9	-	-	-	-	-	-	24 weeks
Tian et al. (2017)	58 (33/25)	58 (30/28)	IGU 25 mg Bid + MTX 10 mg once or twice a week	MTX 10 mg once or twice a week	DAS28, number of tender joints, number of swollen joints, TNF-α, β-CTX, T-PINP, N-MID, 25(OH)D, ESR, CRP, adverse events	52.6 ± 7.6	49.7 ± 8.4	9.4 ± 2.3	8.7 ± 2.1	-	-	-	-	-	-	24 weeks
Chen et al. (2018)	60 (40/20)	60 (38/22)	IGU 25 mg Bid + MTX 10 mg once a week	MTX 10 mg once a week	Morning stiffness time, number of tender joints, number of swollen joints,TNF-α, β-CTX, T-PINP, N-MID, 25(OH)D, CRP, adverse events	45.7 ± 5.4	45.9 ± 4.8	7.2 ± 2.0	6.9 ± 2.1	34.48 ± 4.07	37.96 ± 4.01	-	-	-	-	24 weeks
Xiong et al. (2015)	30 (24/6)	28 (21/7)	IGU 25 mg Bid + MTX 10 mg once a week	MTX 10 mg once a week	CCP, RF, CRP, ESR, DAS28, adverse events	56 ± 12	51 ± 13	4.83 ± 4.42		43 ± 9	41 ± 8	81 ± 15	83 ± 16	6.8 ± 0.7	6.7 ± 0.7	24 weeks
Mo et al. (2015)	30 (22/8)	30 (21/9)	IGU 25 mg Bid + MTX 15 mg once a week	MTX 15 mg once a week	ACR20, ACR50, ACR70, ESR, CRP, RF, CCP, adverse events	31.8 ± 8.5	31.9 ± 8.6	5.6 ± 1.8	5.5 ± 1.9	92.38 ± 38.29	90.28 ± 33.85	86.26 ± 35.82	85.63 ± 35.26	-	-	12 weeks
Fan et al. (2020)	38 (20/18)	37 (21/16)	IGU 25 mg Bid + MTX 10 mg once a week at the beginning; 12.5 mg once a week after 2 weeks; 15 mg once a week after 4 weeks	MTX 10 mg once a week at the beginning; 12.5 mg once a week after 2 weeks; 15 mg once a week after 4 weeks	Morning stiffness time, number of tender joints, number of swollen joints, DAS28, TNF-α, β-CTX, T-PINP, N-MID, 25(OH)D	49.0 ± 10.1	48.7 ± 10.2	1.29 ± 0.30	1.28 ± 0.26	-	-	-	-	7.26 ± 0.19	7.31 ± 0.28	24 weeks
Zhao et al. (2018)	36 (24/12)	36 (23/13)	IGU 25 mg Bid + MTX 7.5 mg once a week	MTX 7.5 mg once a week	Morning stiffness time, DAS28, CRP, adverse events	47.20 ± 3.40	50.80 ± 4.10	4.28 ± 0.36	3.91 ± 0.3	57.8 ± 12.4	58.3 ± 12.2	68.5 ± 9.7	69.2 ± 10.8	6.9 ± 2.8	6.8 ± 2.9	12 weeks
Li et al. (2020)	20 (17/3)	13 (11/2)	IGU 25 mg Bid + MTX 10 mg once a week	MTX 10 mg once a week + adalimumab 40 mg once every 2 weeks	DAS28	58 ± 11	55 ± 11	5.17 ± 1.67	5 ± 1.5	46 (39, 89)*	46 (43.5, 81.5)*	13.2 (2.5, 50)*	15.3 (12.2, 45.4)*	4.92 ± 1.10	4.13 ± 0.90	24 weeks
Li et al. (2019)	51 (26/25)	51 (24/27)	IGU 25 mg Bid + MTX 15 mg once a week	MTX 15 mg once a week	VEGF, TNF-α, adverse events	74.16 ± 2.42	74.32 ± 2.52	5.38 ± 0.62	5.41 ± 0.60	-	-	-	-	-	-	15 weeks
Meng et al. (2017)	60 (32/28)	60 (35/35)	IGU 25 mg Bid + MTX 10 mg once a week	MTX 10 mg once a week	RF, CRP, number of swollen joints, number of tender joints, adverse events	64.83 ± 9.41	64.31 ± 8.22	5.64 ± 2.41	6.22 ± 2.73	14.25 ± 3.23	14.76 ± 3.15	-	-	-	-	12 weeks
Li et al. (2016)	44 (26/18)	40 (24/16)	IGU 25 mg Qd + MTX 7.5–10 mg once a week	MTX 7.5–10 mg once a week + tripterygium glycosides 20 mg Bid	DAS28, morning stiffness time, number of swollen joints, number of tender joints, ESR, CRP, adverse events	60–77	60–82	0.17–1.83	0.25–2	20 ± 26	20 ± 29	48 ± 42	43 ± 36	6.5 ± 4.8	6.4 ± 4.6	12 weeks
Xia et al. (2020)	50 (39/11)	50 (37/13)	IGU 25 mg Bid + MTX 7.5 mg once a week at the beginning, increase by 2.5 mg per week, with a final dose of 15 mg	MTX 7.5 mg once a week at the beginning, increase by 2.5 mg per week, with a final dose of 15 mg + tripterygium glycosides 1–1.5 mg/kg	Number of tender joints, number of swollen joints, ESR, CRP	53.73 ± 2.78	53.62 ± 2.45	4.20 ± 1.41	4.17 ± 1.22	15.38 ± 1.31	15.24 ± 1.52	53.49 ± 8.77	53.26 ± 8.41	-	-	12 weeks
Meng et al. (2015)	33 (29/4)	33 (26/7)	IGU 25 mg Bid + MTX 10 mg once a week	MTX 10 mg once a week + leflunomide 10 mg Qd	DAS28, ACR20, ACR50, ACR70, adverse events	44.2 ± 20.5	41.7 ± 22.8	-	-	-	-	-	-	6.53 ± 1.65	6.37 ± 1.89	16 weeks
Shi et al. (2015)	30 (22/8)	30 (20/10)	IGU 25 mg Bid + MTX 10 mg once a week at the beginning; 12.5 mg twice a week after 4 weeks	MTX 10 mg once a week at the beginning; 12.5 mg twice a week after 4 weeks	Number of tender joints, number of swollen joints, DAS28, HAQ, ESR, CRP, ACR20, ACR50, ACR70, adverse events	48.9 ± 12.2	48.4 ± 10.2	7.5 ± 4.8	7.1 ± 6.6	33.5 ± 35.5	27.5 ± 27.5	57.7 ± 37.7	57.9 ± 28.9	5.2 ± 1.3	5.2 ± 0.9	24 weeks
Mo et al. (2018)	30 (22/8)	30 (24/6)	IGU 25 mg Bid + MTX 10 mg once a week	MTX 10 mg once a week + tripterygium glycosides 20 mg Bid	DAS28, ESR, CRP, CCP, RF, adverse events	45 ± 11.6	43.3 ± 10.25	0.75 ± 0.58	0.82 ± 0.54	45.36 ± 20.32	42.65 ± 19.65	90.68 ± 48.68	86.78 ± 42.56	6.78 ± 1.55	6.65 ± 1.78	12 weeks
Meng et al. (2016)	30 (26/4)	30 (27/3)	IGU 25 mg Bid + MTX 15 mg once a week	MTX 15 mg once a week	DAS28, VEGF, TNF-α, adverse events	41.6 ± 20.3	45.1 ± 19.2	-	-	-	-	-	-	5.97 ± 1.62	6.40 ± 1.90	16 weeks
Wang et al. (2019)	47 (25/22)	46 (23/23)	IGU 25 mg Bid + MTX 15 mg once a week	MTX 15 mg once a week	TNF-α, CRP, RF, ESR, DAS28, morning stiffness time, number of tender joints, number of swollen joints	48.13 ± 6.40	47.83 ± 6.37	5.60 ± 0.70	5.41 ± 0.72	73.25 ± 10.11	73.28 ± 10.09	64.30 ± 9.01	64.28 ± 9.05	6.30 ± 0.88	6.27 ± 0.85	24 weeks
Qi et al. (2019)	40 (not known)	40 (not known)	IGU 25 mg Bid + MTX 7.5 mg once a week at the beginning, gradually increase to 10 mg within 4 weeks	MTX 7.5 mg once a week at the beginning, gradually increase to 10 mg within 4 weeks	HAQ, number of tender joints, number of swollen joints, ACR20, ACR50, ACR70, ESR, CRP, CCP, adverse events	25–65	-	-	-	33.6 ± 13.1	34.0 ± 15.2	51.9 ± 11.3	56.3 ± 14.6	-	-	24 weeks
Ju et al. (2020)	58 (25/33)	58 (23/35)	IGU 25 mg Bid + MTX 10 mg once a week	MTX 10 mg once a week	DAS28, ESR, CRP, RF, VEGF, TNF-α	42.31 ± 13.78	41.87 ± 13.94	4.56 ± 0.58	4.72 ± 0.43	49.12 ± 13.02	47.94 ± 12.72	69.76 ± 18.50	69.32 ± 17.98	6.46 ± 2.24	6.27 ± 2.12	24 weeks
Zhao et al. (2016)	30 (19/11)	30 (21/9)	IGU 25 mg Bid + MTX 10 mg once a week	MTX 15 mg once a week	ACR20, ACR50, ACR70, adverse events	30.1 ± 2.4	28.1 ± 3.4	6.9 ± 1.1	5.7 ± 3.8	-	-	-	-	-	-	24 weeks
Duan et al. (2015)	30 (22/8)	30 (20/10)	IGU 25 mg Bid + MTX 10 mg once a week at the beginning, gradually increase to 12.5 mg within 4 weeks	MTX 10 mg once a week at the beginning, gradually increase to 12.5 mg within 4 weeks	number of tender joints, number of swollen joints, ESR, CRP, DAS28, HAQ, adverse events	48.9 ± 12.2	48.4 ± 10.2	7.5 ± 4.8	7.1 ± 6.6	33.5 ± 35.5	27.5 ± 27.5	57.7 ± 37.7	57.9 ± 28.9	5.2 ± 1.3	5.2 ± 0.9	24 weeks
Hara et al. (2014)	164 (134/30)	68 (52/16)	IGU 25 mg Qd for the first 4 weeks of the extension period 25 mg Bid for the subsequent 20 week + MTX 6–8 mg once a week	MTX 6–8 mg once a week + placebo	ACR20, ACR50, ACR70, number of tender joints, number of swollen joints, HAQ, CRP, ESR, RF, DAS28, adverse events	54.8 ± 9.9	53.5 ± 10.0	4.48 ± 0.83	4.48 ± 0.88	18.4 ± 19.4	17.1 ± 16.1	45.6 ± 21.0	40.1 ± 23.2	4.87 ± 0.89	4.96 ± 0.87	24 weeks
Xia et al. (2016)	131 (107/24)		IGU 25 mg Bid + MTX 10 mg once a week	MTX 10 mg once a week	Morning stiffness time, number of tender joints, number of swollen joints, ESR, CRP, HAQ	46.63 ± 10.61	-	-	-	38.68 ± 6.25	44.86 ± 4.80	69.39 ± 3.95	62.34 ± 4.08	4.55 ± 0.09	4.70 ± 0.10	24 weeks

### Risk of Bias of Included Studies

#### Sequence Generation and Allocation Concealment

Fifteen RCTs describe the random sequence generating method: 10 RCTs utilized random number table (Meng et al., 2015; Mo et al., 2015; Shi et al., 2015; Zhao et al., 2016; Tian et al., 2017; Chen et al., 2018; Mo et al., 2018; Ju et al., 2020; Xia et al., 2020; Xiong et al., 2020); two RCT utilized the two-color ball method (Zhao et al., 2018; Li et al., 2020); Fan et al. utilized the odd and even number random method; Xu et al. utilized random lottery; Li et al. (2019) utilized the envelope random method; and Tian et al. utilized centralized randomization. We rate these studies as low risk of bias. The other RCTs did not describe the random sequence generating methods and were rated as unclear risk of bias. Tian et al. (2020) used the “double dummy” method to make the number and appearance of the tablets in the two groups similar. We consider this to be allocation concealment and rated it as low risk of bias. The other RCTs did not state whether allocation concealment was carried out, so we evaluated the risk of bias as unclear.

#### Blinding

Tian et al. (2020) and Hara et al. (2014) used a double-blind method, so they were considered to be a low risk of bias. Although Ding et al. (2019) and Meng et al. (2017) did not state whether blinding was used, but because its outcomes are objective indicators (β-CTX, N-MID, T-PINP), which is less affected by blinding, we assessed the risk of bias as low. Other RCTs did not mention whether to use blinding, and their main outcome indicators are subjective evaluation indicators (such as DAS28, ACR20, ACR50, ACR70), which are easily affected by not implementing the blind method. Therefore, we evaluate them as a high risk of bias.

#### Incomplete Outcome Data and Selective Reporting

Li et al. (2020) and Xia et al. (2016) have incomplete outcome, and there is an imbalance between the number of missing persons and the reasons for the missing between groups. Hence, they were rated as having a high risk of bias. The other RCTs do not have incomplete outcome data. Mo et al. (2015) mentioned the morning stiffness time, number of tender joints, and number of swollen joints but did not report in the article. Therefore, we thought that they have selective reporting and assess the risk of bias as high. No selective reports were found in other RCTs, so they were considered low risk of bias.

#### Other Potential Bias

**FIGURE 2:**
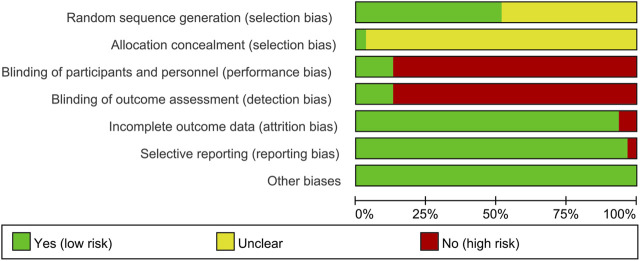
Risk-of-bias graph.

**FIGURE 3:**
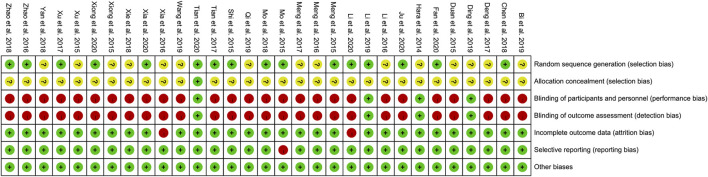
Risk-of-bias summary.

Other sources of bias were not observed in 13 RCTs; therefore, the risks of other bias of the RCTs were low (Figures 2, 3).

### Primary Outcomes

#### ACR20

**FIGURE 4:**
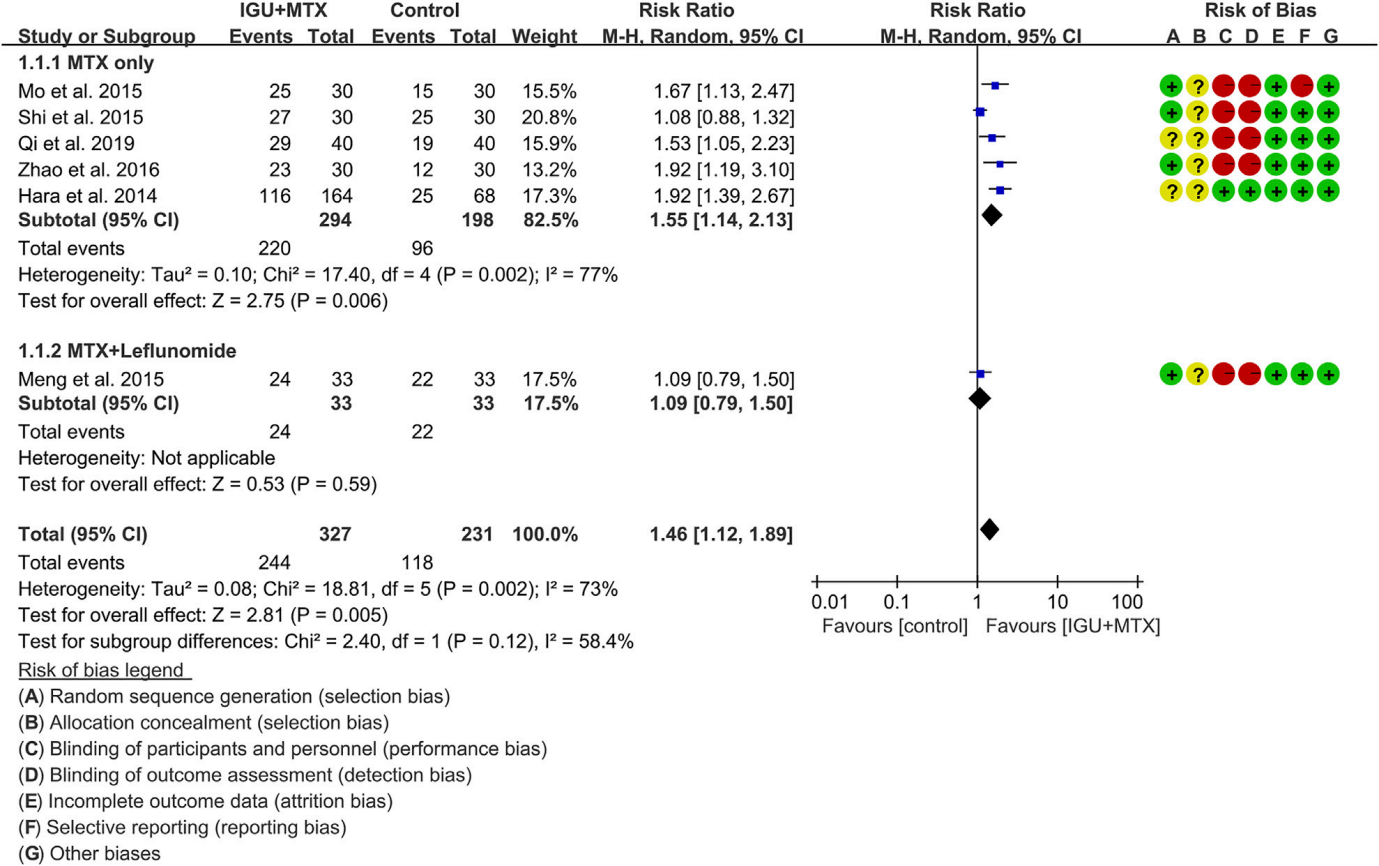
The results of ACR20.

Six RCTs assessed the ACR20 of patients, which involves 327 patients in the IGU + MTX group and 231 patients in the control group. According to the intervention characteristics, six RCTs were subdivided into two subgroups (MTX-only subgroup and MTX + leflunomide subgroup). There was high heterogeneity in each subgroup (MTX only: I^2^ = 77%, *p* = 0.002; MTX + leflunomide: not applicable) among the RCTs. The random-effect model was used. According to Figure 4, the ACR20 in the IGU + MTX group was higher than that in the control group in the MTX-only subgroup (RR 1.55, 95% CI 1.14–2.13, *p* = 0.006; random-effect model), but its difference is of no statistical significance in the MTX + leflunomide subgroup (RR 1.09, 95% CI 0.79–1.89, *p* = 0.59; random-effect model). The summary result also showed that the ACR20 in the IGU + MTX group was higher (RR 1.46, 95% CI 1.12–1.89, *p* = 0.005; random-effect model) (Figure 4).

#### ACR50

**FIGURE 5:**
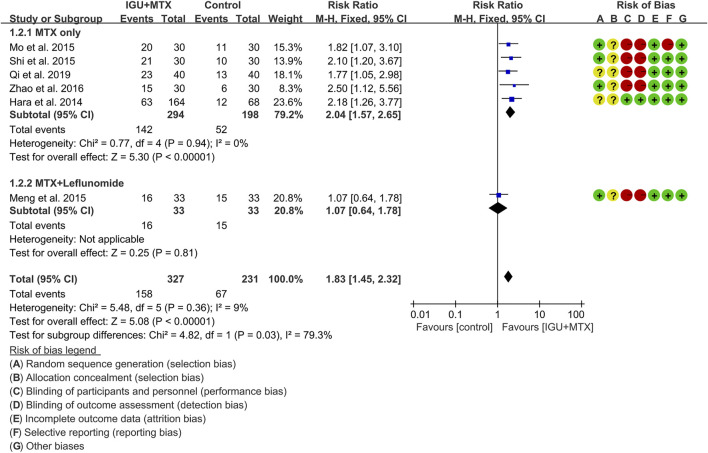
The results of ACR50.

Six RCTs assessed the ACR50 of patients, which involves 327 patients in the IGU + MTX group and 231 patients in the control group. According to the intervention characteristics, they were subdivided into two subgroups (MTX-only subgroup and MTX + leflunomide subgroup). There was low heterogeneity in each subgroup (MTX only: I^2^ = 0%, *p* = 0.94; MTX + leflunomide: not applicable) among the RCTs. The fixed-effect model was used. According to Figure 5, the ACR50 in the IGU + MTX group was higher than that in the control group in the MTX-only subgroup (RR 2.04, 95% CI 1.57–2.65, *p* < 0.00001; fixed-effect model), but its difference is of no statistical significance in the MTX + leflunomide subgroup (RR 1.07, 95% CI 0.64–1.78, *p* = 0.81; fixed-effect model). The summary result also showed that the ACR50 in the IGU + MTX group was higher (RR 1.83, 95% CI 1.45–2.32, *p* < 0.00001; fixed-effect model) (Figure 5).

#### ACR70

**FIGURE 6:**
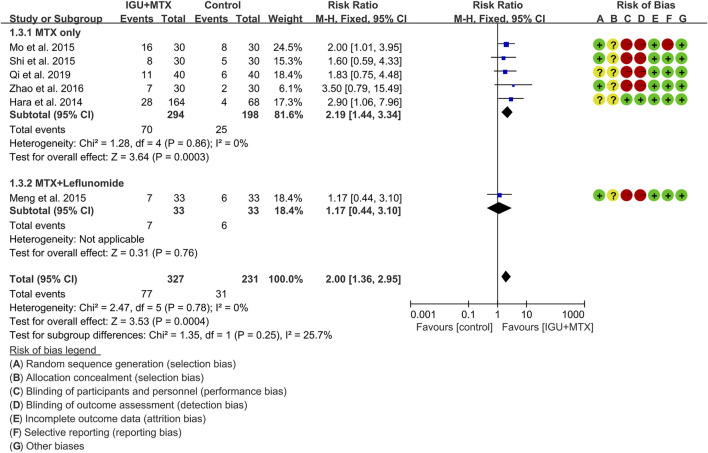
The results of ACR70.

Six RCTs assessed the ACR70 of patients, which involves 327 patients in the IGU + MTX group and 231 patients in the control group. According to the intervention characteristics, they were subdivided into two subgroups (MTX-only subgroup and MTX + leflunomide subgroup). There was low heterogeneity in each subgroup (MTX only: I^2^ = 0%, *p* = 0.86; MTX + leflunomide: not applicable) among the RCTs. The fixed-effect model was used. According to Figure 6, the ACR70 in the IGU + MTX group was higher than that in the control group in the MTX-only subgroup (RR 2.19, 95% CI 1.44–3.34, *p* = 0.00003; fixed-effect model), but its difference is of no statistical significance in the MTX + leflunomide subgroup (RR 1.17, 95% CI 0.44–3.10, *p* = 0.76; fixed-effect model). The summary result also showed that the ACR70 in the IGU + MTX group was higher (RR 2.00, 95% CI 1.36–2.95, *p* = 0.00004; fixed-effect model) (Figure 6).

#### DAS28

**FIGURE 7:**
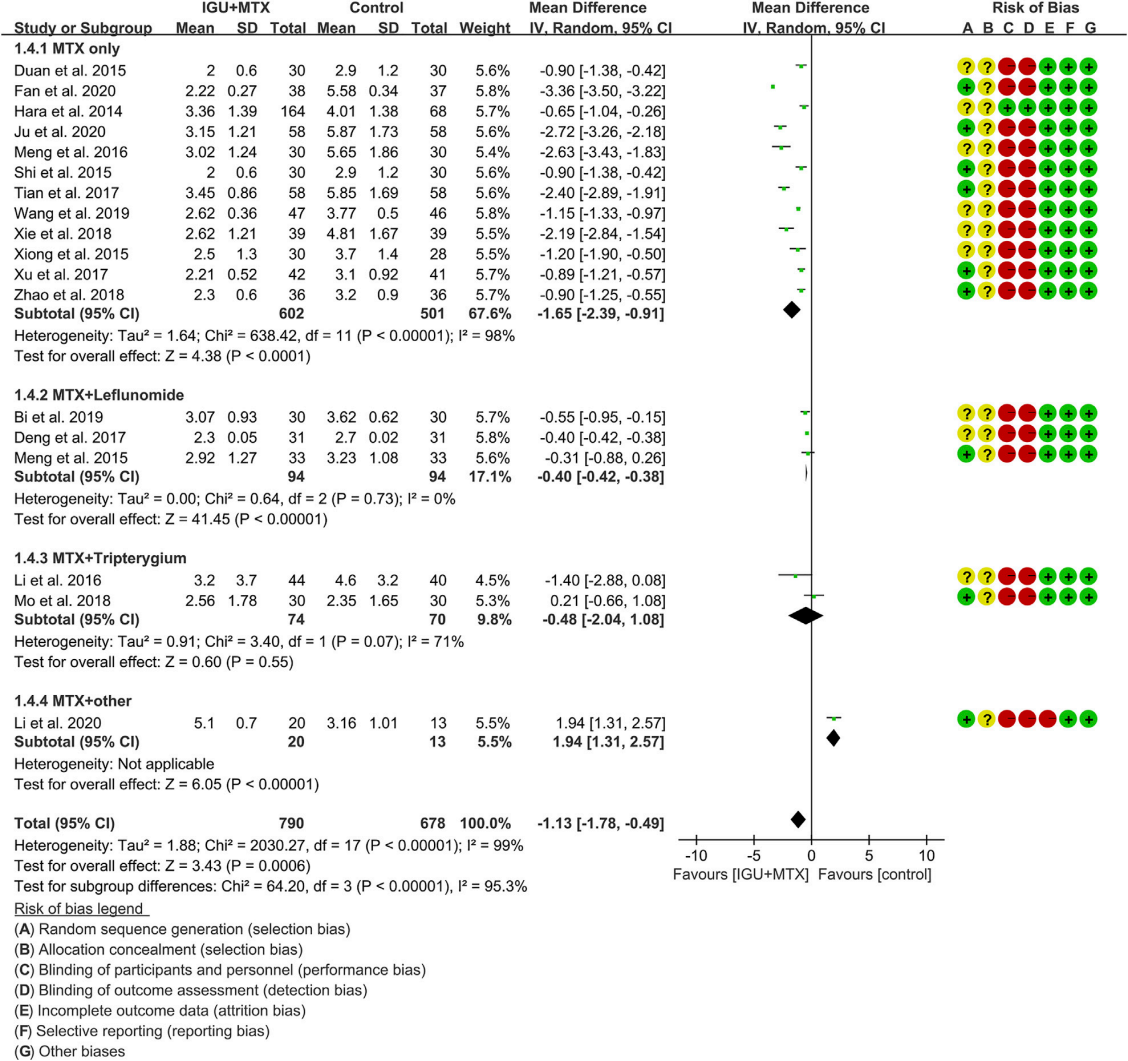
The results of DAS28.

Eighteen RCTs assessed the DAS28 of patients, which involves 790 patients in the IGU + MTX group and 678 patients in the control group. According to the intervention characteristics, they were subdivided into four subgroups (MTX-only subgroup, MTX + leflunomide subgroup, MTX + tripterygium subgroup, MTX + other subgroup). There was high heterogeneity in the most subgroup (MTX only: I^2^ = 98%, *p* < 0.00001; MTX + leflunomide: I^2^ = 0%, *p* = 0.73; MTX + tripterygium: I^2^ = 71%, *p* = 0.07; MTX + other: not applicable) among the RCTs. The random-effect model was used. According to Figure 7, the DAS28 in the IGU + MTX group was lower than that in the control group in the MTX-only subgroup (WMD -1.65, 95% CI −2.39 to −0.91, *p* < 0.0001; random-effect model) and MTX + leflunomide subgroup (WMD −0.40, 95% CI −0.42 to −0.38, *p* < 0.0001; random-effect model), but its difference is of no statistical significance in the MTX + tripterygium subgroup (WMD −0.48, 95% CI −2.04 to 1.08, *p* = 0.55; random-effect model). However, in the MTX + other subgroup, the DAS28 in IGU + MTX group is higher than that of the control group (WMD 1.94, 95% CI 1.31–2.57, *p* < 0.00001; random-effect model). The summary result also showed that the DAS28 in the IGU + MTX group was lower (WMD −1.13, 95% CI −1.78 to −0.49, *p* = 0.0006; random-effect model).

### Secondary Outcomes

The secondary outcomes include 1) symptom-related outcomes: HAQ, morning stiffness time (min), number of swollen joints, and number of tender joints; 2) bone protection-related outcomes: N-MID, total T-PINP levels, 25(OH)D, and β-CTX; 3) inflammation and immune response-related outcomes: RF, ESR, CRP, anti-CCP, and TNF-α; and 4) angiogenesis-related outcomes: VEGF. The results are shown in Table 2.

**TABLE 2:** The results of secondary outcomes.

Outcomes	Subgroup	Effect				Heterogeneity test			Statistical method	Studies (N)	Sample size (N)	Figures
		MD	95% CI	*p*	Significance	Tau^2^	I^2^ (%)	*p*				
HAQ	MTX only	−0.28	[−0.44, −0.13]	*p* = 0.0004	Yes	0.02	87	*p* = 0.00001	Random	5	523	Supplementary Figure S1
	MTX + leflunomide	−0.04	[−0.05, −0.03]	*p* < 0.00001	Yes	0	0	*p* = 0.77	Random	2	206	
	Summary	−0.12	[−0.16, −0.07]	*p* < 0.00001	Yes	0	90	*p* < 0.00001	Random	7	729	
Morning stiffness time	MTX only	−2.37 (SMD)	[−3.20, −1.54]	*p* < 0.00001	Yes	1.34	95	*p* < 0.00001	Random	8	716	Supplementary Figure S2
	MTX + tripterygium	−0.89 (SMD)	[−1.34, −0.44]	*p* = 0.0001	Yes	-	-	-	Random	1	84	
	Summary	−2.2 (SMD)	[−2.96, −1.44]	*p* < 0.00001	Yes	1.28	95	*p* < 0.00001	Random	9	800	
Number of tender joints	MTX only	−2.47	[−3.18, −1.77]	*p* < 0.00001	Yes	1.36	97	*p* < 0.00001	Random	12	1,229	Supplementary Figure S3
	MTX + leflunomide	−2	[−5.02, 1.02]	*p* = 0.19	No	-	-	-	Random	1	144	
	MTX + tripterygium	−2.06	[−2.68, −1.43]	*p* < 0.00001	Yes	0	0	*p* = 0.71	Random	2	184	
	Summary	−2.41	[−3.05, −1.76]	*p* < 0.00001	Yes	1.3	96	*p* < 0.00001	Random	15	1,557	
Number of swollen joints	MTX only	−2.45	[−3.24, −1.67]	*p* < 0.00001	Yes	1.77	99	*p* < 0.00001	Random	12	1,229	Supplementary Figure S4
	MTX + leflunomide	0	[−3.07, 3.07]	*p* = 1.00	No	-	-	-	Random	1	144	
	MTX + tripterygium	−1.81	[−2.35, −0.87]	*p* < 0.001	Yes	0	0	*p* = 0.44	Random	2	184	
	Summary	−2.26	[−2.99, −1.54]	*p* < 0.00001	Yes	1.74	98	*p* < 0.00001	Random	15	1,557	
N-MID	MTX only	4.05	[3.26, 4.83]	*p* < 0.00001	Yes	0.54	62	*p* = 0.02	Random	6	559	Supplementary Figure S5
T-PINP	MTX only	10.92	[9.09, 12.75]	*p* < 0.00001	Yes	3.87	80	*p* = 0.0002	Random	6	559	Supplementary Figure S6
β-CTX	MTX only	−0.29	[−0.35, −0.23]	*p* < 0.00001	Yes	0	81	*p* = 0.0003	Random	5	457	Supplementary Figure S7
25(OH)D	MTX only	2.83	[1.62, 4.04]	*p* < 0.00001	Yes	1.47	84	*p* < 0.0001	Random	5	457	Supplementary Figure S8
RF	MTX only	−2.06 (SMD)	[−3.19, −0.93]	*p* < 0.00001	Yes	2.25	97	*p* < 0.00001	Random	7	757	Supplementary Figure S9
	MTX + leflunomide	−0.02 (SMD)	[−0.36, 0.32]	*p* = 0.89	No	0.03	40	*p* = 0.20	Random	2	302	
	MTX + tripterygium	−2.17 (SMD)	[−2.82, −1.53]	*p* < 0.00001	Yes	-	-	-	Random	1	60	
	Summary	−1.65 (SMD)	[−2.48, −0.82]	*p* < 0.0001	Yes	1.71	97	*p* < 0.00001	Random	10	1,119	
ESR	MTX only	−13.88	[−16.97,−10.79]	*p* < 0.00001	Yes	25.39	94	*p* < 0.00001	Random	13	1,201	Supplementary Figure S10
	MTX + leflunomide	−0.08	[−9.65, 9.49]	*p* = 0.99	No	30.4	61	*p* = 0.11	Random	2	194	
	MTX + tripterygium	−8.06	[−10.79, −5.34]	*p* < 0.00001	Yes	1.29	18	*p* = 0.30	Random	3	244	
	Summary	−11.59	[−14.38, −8.80]	*p* < 0.00001	Yes	28.58	94	*p* < 0.00001	Random	18	1,639	
CRP	MTX only	−2.18 (SMD)	[−2.95, −1.42]	*p* < 0.00001	Yes	2.16	97	*p* < 0.00001	Random	15	1,441	Supplementary Figure S11
	MTX + leflunomide	−0.43 (SMD)	[−1.48, 0.62]	*p* = 0.42	No	0.52	91	*p* = 0.0008	Random	2	197	
	MTX + tripterygium	−0.7 (SMD)	[−0.96,−0.44]	*p* < 0.00001	Yes	0	0	*p* = 0.45	Random	3	244	
	Summary	−1.76 (SMD)	[−2.34, −1.18]	*p* < 0.00001	Yes	1.65	96	*p* < 0.00001	Random	20	1882	
Anti-CCP	MTX only	−17.61	[−22.64, −12.57]	*p* < 0.00001	Yes	0.17	0	*p* = 0.92	Fixed	3	178	Supplementary Figure S12
	MTX + leflunomide	−27.64	[−49.83, −5.45]	*p* = 0.01	Yes	1.82	45	*p* = 0.18	Fixed	2	183	
	MTX + tripterygium	−2.33	[−4.85, 0.19]	*p* = 0.07	No	-	-	-	Fixed	1	60	
	Summary	−5.61	[−7.86, −3.37]	*p* < 0.00001	Yes	34.06	85	*p* < 0.00001	Fixed	6	421	
TNF-α	MTX only	−2.35 (SMD)	[−3.19, −1.50]	*p* < 0.00001	Yes	1.58	96	*p* < 0.00001	Random	9	830	Supplementary Figure S13
VEGF	MTX only	−3.22 (SMD)	[−4.95, −1.48]	*p* = 0.0003	Yes	3.7	98	*p* < 0.00001	Random	5	416	Supplementary Figure S14

### Adverse Events

**FIGURE 8:**
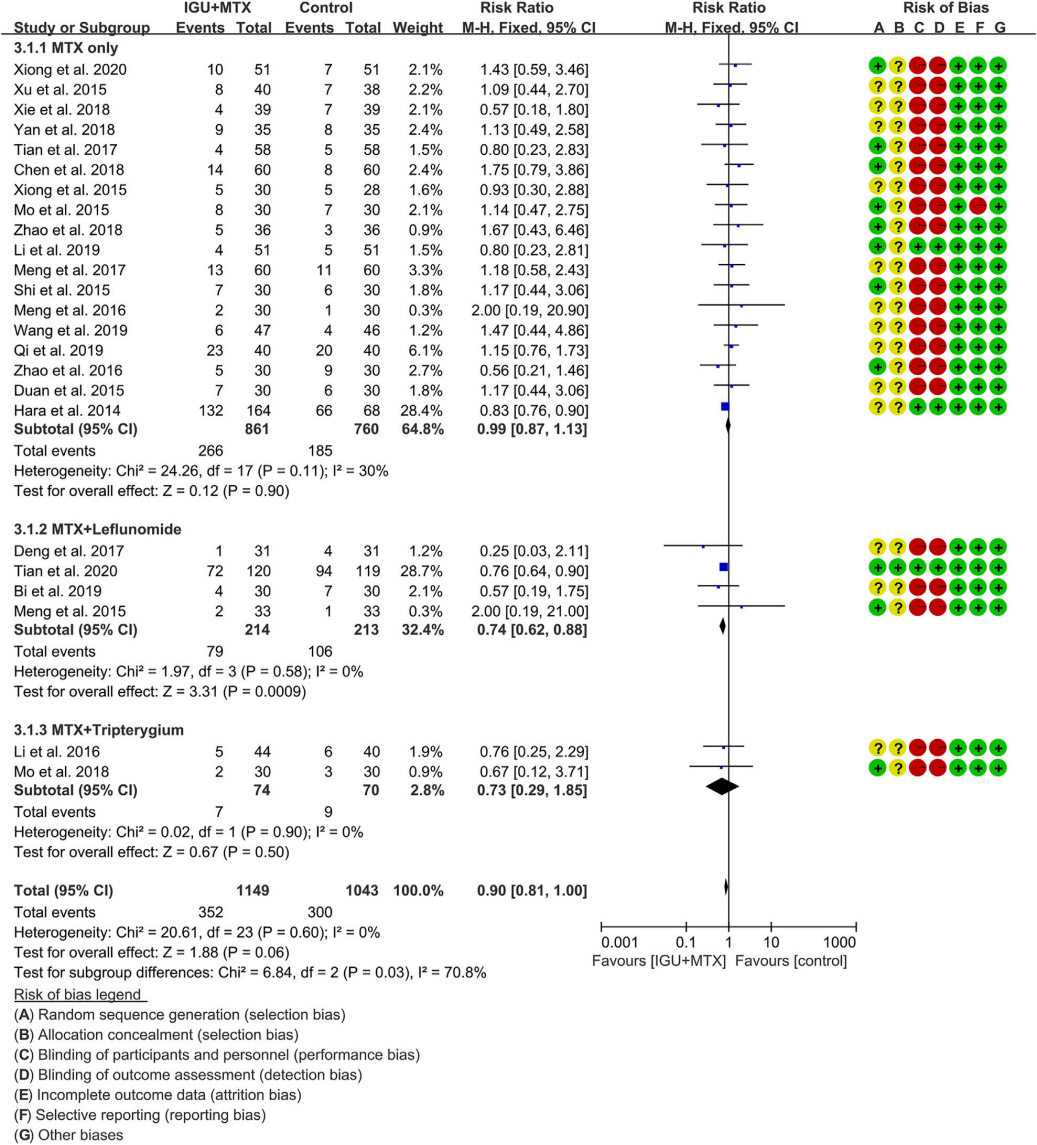
Adverse events.

Twenty-four RCTs reported the adverse events. According to the intervention characteristics, they were subdivided into three subgroups (MTX-only subgroup, MTX + leflunomide subgroup, MTX + tripterygium subgroup). There was low heterogeneity in the most subgroup (MTX only: I^2^ = 30%, *p* = 0.11; MTX + leflunomide: I^2^ = 0%, *p* = 0.58; MTX + tripterygium: I^2^ = 0%, *p* = 0.90) among the RCTs. The fixed-effect model was used. According to Figure 8, the adverse events in the IGU + MTX group were lower than those in the control in the MTX + leflunomide subgroup (RR 0.74, 95% CI 0.62–0.88, *p* = 0.0009; fixed-effect model). However, the difference is of no statistical significance in the MTX-only subgroup (RR 0.99, 95% CI 0.87–1.13, *p* = 0.90; fixed-effect model) and MTX + tripterygium subgroup (RR 0.73, 95% CI 0.29–1.85, *p* = 0.50; fixed-effect model). The summary result also showed that the difference is of no statistical significance between the two groups (RR 0.90, 95% CI 0.81–1.00, *p* = 0.06; fixed-effect model).

### Other Subgroup Analysis Results

This study also conducted a subgroup analysis of primary outcomes based on duration and drug dose (Table 3). For the drug dose, since the dose of IGU is basically the same, the subgroup analysis is performed based on the starting dose of MTX. The results show that there is no obvious rule for the outcomes of different starting doses. For the duration of the intervention, the results showed that DAS28 improved after the intervention lasted at least 12 weeks.

**TABLE 3:** Other subgroup analysis results.

Outcomes	Subgroup	Effect				Heterogeneity test			Statistical method	Studies (N)	Sample size (N)
		MD	95% CI	*p*	Significance	Tau^2^	I^2^ (%)	*p*			
ACR20-duration	12 weeks	1.67	[1.13, 2.47]	*p* = 0.01	Yes	-	-	-	Random	1	60
	16 weeks	1.09	[0.79, 1.50]	*p* = 0.59	No	-	-	-	Random	1	66
	24 weeks	1.46	[1.04, 1.89]	*p* = 0.03	Yes	0.13	82	*p* = 0.0007	Random	4	432
ACR50-duration	12 weeks	1.82	[1.07, 3.10]	*p* = 0.03	Yes	-	-	-	Fixed	1	60
	16 weeks	1.07	[0.64, 1.78]	*p* = 0.81	No	-	-	-	Fixed	1	66
	24 weeks	2.09	[1.55, 2.81]	*p* < 0.00001	Yes	-	0	*p* = 0.89	Fixed	4	432
ACR70-duration	12 weeks	2	[1.01, 3.95]	*p* = 0.05	Yes	-	-	-	Fixed	1	60
	16 weeks	1.17	[0.44, 3.10]	*p* = 0.76	No	-	-	-	Fixed	1	66
	24 weeks	2.27	[1.35, 3.84]	*p* = 0.002	Yes	-	0	*p* = 0.74	Fixed	4	432
DAS28-duration	12 weeks	−0.62	[−1.07, −0.17]	*p* = 0.007	Yes	0.11	56	*p* = 0.08	Random	4	276
	16 weeks	−1.69	[−3.15, −0.23]	*p* = 0.02	Yes	1.54	93	*p* < 0.00001	Random	3	204
	24 weeks	−1.27	[−2.28, −0.26]	*p* = 0.01	Yes	2.34	99	*p* < 0.00001	Random	9	843
	48 weeks	−0.62	[−1.09, −0.14]	*p* = 0.01	Yes	0.11	89	*p* = 0.003	Random	2	145
Adverse event-duration	12 weeks	0.99	[0.65, 1.49]	*p* = 0.95	No	-	0	*p* = 0.81	Fixed	6	456
	15 weeks	0.8	[0.23, 2.81]	*p* = 0.73	No	-	-	-	Fixed	1	102
	16 weeks	0.89	[0.36, 2.19]	*p* = 0.80	No	-	0	*p* = 0.48	Fixed	3	204
	24 weeks	0.97	[0.85, 1.12]	*p* = 0.71	No	-	48	*p* = 0.04	Fixed	11	1,051
	48 weeks	0.79	[0.35, 1.77]	*p* = 0.56	No	-	37	*p* = 0.21	Fixed	2	140
	52 weeks	0.76	[0.64, 0.90]	*p* = 0.002	Yes	-	-	-	Fixed	1	239
ACR20-dosage	MTX starts from 7.5 mg	1.53	[1.05, 2.23]	*p* = 0.03	Yes	-	-	-	Random	1	80
	MTX starts from 10 mg	1.24	[0.90, 1.70]	*p* = 0.18	No	0.05	66	*p* = 0.05	Random	3	186
	MTX starts from 15 mg	1.67	[1.13, 2.47]	*p* = 0.01	Yes	-	-	-	Random	1	60
ACR50-dosage	MTX starts from 7.5 mg	1.77	[1.05, 2.98]	*p* = 0.03	Yes	-	-	-	Fixed	1	80
	MTX starts from 10 mg	1.68	[1.19, 2.36]	*p* = 0.003	Yes	-	56	*p* = 0.10	Fixed	3	186
	MTX starts from 15 mg	1.82	[1.07, 3.10]	*p* = 0.03	Yes	-	-	-	Fixed	1	60
ACR70-dosage	MTX starts from 7.5 mg	1.83	[0.75, 4.48]	*p* = 0.18	No	-	-	-	Fixed	1	80
	MTX starts from 10 mg	1.69	[0.91, 3.15]	*p* = 0.10	No	-	0	*p* = 0.48	Fixed	3	186
	MTX starts from 15 mg	2	[1.01, 3.95]	*p* = 0.05	Yes	-	-	-	Fixed	1	60
DAS28-dosage	MTX starts from 7.5 mg	−0.9	[−1.25, −0.55]	*p* < 0.00001	Yes	-	-	-	Random	1	72
	MTX starts from 10 mg	−0.95	[−1.99, 0.09]	*p* = 0.07	No	3.04	99	*p* < 0.00001	Random	11	728
	MTX starts from 15 mg	−1.84	[−3.28, −0.39]	*p* = 0.01	Yes	1.01	92	*p* = 0.0004	Random	2	153
Adverse event-dosage	MTX starts from 7.5 mg	1.22	[0.81, 1.82]	*p* = 0.34	No	-	0	*p* = 0.60	Fixed	2	153
	MTX starts from 10 mg	0.89	[0.76, 1.04]	*p* = 0.15	No	-	0	*p* = 0.46	Fixed	14	1,215
	MTX starts from 15 mg	1.07	[0.52, 2.20]	*p* = 0.86	No	-	0	*p* = 0.79	Fixed	4	315

### Publication Bias Detection

**FIGURE 9:**
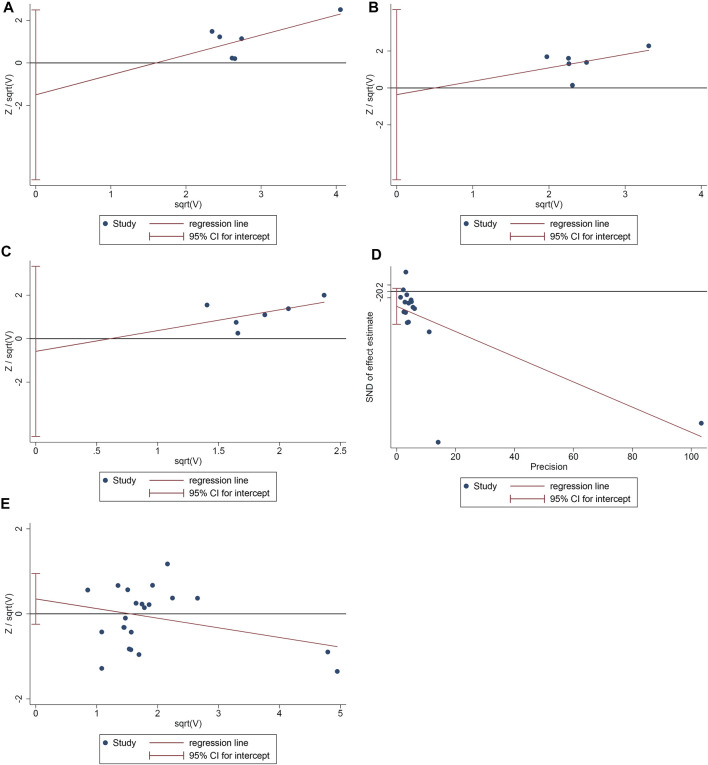
The results of publication bias detection (**A**: ACR20; **B**: ACR50; **C**: ACR70; **D**: DAS28; **E**: adverse events).

The publication bias of the primary outcomes was detected by STATA 15.0. 1) ACR20: the publication bias detection suggests that the possibility of publication bias was small (*p* = 0.355) (Figure 9A). 2) ACR50: the publication bias detection suggests that the possibility of publication bias was small (*p* = 0.837) (Figure 9B). 3) ACR70: the publication bias detection suggests that the possibility of publication bias was small (*p* = 0.699) (Figure 9C). 4) DAS28: the publication bias detection suggests that there may be publication bias (*p* = 0.097) (Figure 9D). 4) Adverse events: the publication bias detection suggests that the possibility of publication bias was small (*p* = 0.234) (Figure 9E).

### Evidence Quality Assessment

The evidence was judged to be high to very low (Table 2). The quality of ACR70 was high; the quality of adverse events and ACR50 test was moderate; the quality of ACR20 was low; and the quality of DAS28 was very low (Table 4).

**TABLE 4:** Summary of findings for the main comparison.

IGU + MTX intervention in patients with RA						
Patient or population: patients with RA						
Outcomes	Illustrative comparative risks* (95% CI)		Relative effect (95% CI)	No. of participants (studies)	Quality of the evidence (GRADE)	Comments
	Assumed risk	Corresponding risk				
	Control	Primary outcomes				
ACR20	Study population		RR 1.46 (1.12–1.89)	558 (6 studies)	⊕⊕⊝⊝ low^a^ ^,^ ^b^	
	511 per 1,000	746 per 1,000 (572–965)				
	Moderate					
	488 per 1,000	712 per 1,000 (547–922)				
ACR50	Study population		RR 1.83 (1.45–2.32)	558 (6 studies)	⊕⊕⊕⊝ moderate^a^	
	290 per 1,000	531 per 1,000 (421–673)				
	Moderate					
	329 per 1,000	602 per 1,000 (477–763)				
ACR70	Study population		RR 2 (1.36–2.95)	558 (6 studies)	⊕⊕⊕⊕ high^a^ ^,^ ^c^	
	134 per 1,000	268 per 1,000 (183–396)				
	Moderate					
	158 per 1,000	316 per 1,000 (215–466)				
Adverse events	Study population		RR 0.9 (0.81–1)	2,192 (24 studies)	⊕⊕⊕⊝ moderate^a^	
	288 per 1,000	259 per 1,000 (233–288)				
	Moderate					
	179 per 1,000	161 per 1,000 (145–179)				
DAS28		The mean DAS28 in the intervention groups was		1,468 (18 studies)	⊕⊝⊝⊝ very low^a^ ^,^ ^b^ ^,^ ^d^	
		1.13 lower (1.78–0.49 lower)				

*The basis for the assumed risk (e.g., the median control group risk across studies) is provided in footnotes. The corresponding risk (and its 95% confidence interval) is based on the assumed risk in the comparison group and the relative effect of the intervention (and its 95% CI).

a Downgraded one level due to serious risk of bias (random sequence generation, allocation concealment, blinding, incomplete outcomes) and most of the data comes from the RCTs, with moderate risk of bias.

b Downgraded one level due to the probably substantial heterogeneity.

c Upgraded one level due to the RR ≥ 2.

d Downgraded one level due to the publication bias.

CI, confidence interval; RR, risk ratio.

GRADE Working Group grades of evidence.

High quality: further research is very unlikely to change our confidence in the estimate of effect.

Moderate quality: further research is likely to have an important impact on our confidence in the estimate of effect and may change the estimate.

Low quality: further research is very likely to have an important impact on our confidence in the estimate of effect and is likely to change the estimate.

Very low quality: we are very uncertain about the estimate.

## Discussion

### Main Outcome Summary

This systematic review and meta-analysis included 31 RCTs involving 2,776 participants. The number of study participants was mostly between 30 and 240, and the interventions in the control group were mostly MTX alone. The control group of Deng et al. (2017), Tian et al. (2020), Bi et al. (2019), and Meng et al. (2015) used MTX + leflunomide; the control group of Li et al. (2019), Xia et al. (2020), and Mo et al. (2018) used MTX + tripterygium; and the control group of Li et al. (2020) used MTX + adalimumab. Sixteen RCTs failed to describe their random sequence generating method. Only Tian et al. (2020) described the allocation concealment methods. Twenty-nine RCTs failed to utilize blinding. Li et al. (2020) and Xia et al. (2016) have incomplete outcome, and Mo et al. (2015) mentioned the morning stiffness time, number of tender joints, and number of swollen joints but did not report in the article. The overall risk of bias is higher.

The main findings of this study can be summarized as the following: 1) compared with MTX alone, the ACR20, ACR50, and ACR70 of IGU + MTX are higher, while DAS28 is lower; and the symptom-related outcomes (HAQ, morning stiffness time, number of tender joints, number of swollen joints), bone protection-related outcomes [N-MID, T-PINP, β-CTX, 25(OH)D], inflammatory and immune response-related outcomes (RF, ESR, CRP, anti-CCP, TNF-α), and angiogenesis outcomes (VEGF) in the IGU + MTX group improved better. In terms of safety, there was no significant difference in the incidence of adverse events between the IGU + MTX group and the MTX-only group. 2) Compared with MTX + leflunomide, IGU + MTX has no significant difference in improving ACR20, ACR50, and ACR70, but IGU + MTX improves DAS28, HAQ, and anti-CCP more significantly. In terms of improving number of tender joints, number of swollen joints, RF, ESR, and CRP, the difference between the two groups was not statistically significant. However, the incidence of adverse events in the IGU + MTX group was lower. Our previous RCT also showed that compared with MTX + leflunomide, IGU + MTX has no statistical significance in improving ACR20, ACR50, ACR70, ESR, and CRP (Tian et al., 2020). Our RCT also shows that IGU + MTX is safer than MTX + leflunomide (Tian et al., 2020). 3) Compared with MTX + tripterygium, IGU + MTX has no significant difference in improving DAS28 and anti-CCP, but IGU + MTX improves morning stiffness time, number of tender joints, number of swollen joints, RF, ESR, and CRP more significantly. In terms of safety, there was no significant difference in the incidence of adverse events between the IGU + MTX group and the MTX + tripterygium group. 4) Compared with the IGU + adalimumab group, the DAS28 in the IGU + MTX group is higher. 5) For the duration of the intervention, DAS28 improved after the intervention lasted at least 12 weeks. 6) The publication bias test of the primary outcomes showed that there was no publication bias in ACR20, ACR50, ACR70, and adverse events, while DAS28 may have publication bias.

In summary, this systematic review and meta-analysis provide a new treatment strategy for the combination of IGU and MTX in the treatment of RA. When the intervention method is (IGU 25 mg Bid, MTX 10–25 mg once a week), and the intervention lasts for at least 12 weeks, the curative effect may be achieved without obvious adverse events.

### Applicability of Evidences

RA is a common autoimmune disease characterized by painful and swollen joints, which severely impairs the patient’s physical function and quality of life. The treatment of RA has made great progress in the past 10 years. Relevant therapeutic drugs have been continuously introduced, which has improved the treatment and management of RA patients. In recent years, standard treatment has been used in the management of many chronic diseases (Jin et al., 2020). Especially considering the complex pathogenesis of RA, a single drug often fails to effectively achieve the treatment goal. Therefore, international and domestic guidelines recommend the use of combination therapy when a single DMARD treatment fails to meet the standard (Jiang et al., 2020; Jin et al., 2020). Combination therapy can improve the efficacy and reduce the incidence of adverse reactions, which is the main trend of RA treatment (Xie et al., 2020).

MTX is a first-line drug for the treatment of RA. Its main mechanism of action is as follows: inhibiting the formation of tetrahydrofolate by inhibiting dihydrofolate reductase, blocking DNA synthesis, and achieving anti-inflammatory and anti-immune effects (Lu et al., 2009; Mimori et al., 2019). Recent studies have shown that MTX is the “anchoring drug” in the treatment of most RA patients. The European Federation for the Prevention and Treatment of Rheumatism (EULAR) recommends that the efficacy of MTX be evaluated after 3 months of treatment. If there is no improvement within 3 months of the first treatment or the treatment goal is not reached within 6 months, the treatment plan should be reconsidered (GRADEpro 2015). The 2018 Chinese Rheumatoid Arthritis Diagnosis and Treatment Guidelines also mentioned that if the standard treatment of MTX still fails to reach the target, it is recommended to combine another synthetic DMARD for treatment (Schünemann et al., 2013). However, in clinical practice, there are a variety of prescriptions for combination drugs for the treatment of RA, such as IGU + MTX, MTX + leflunomide, and islammod + diacerein (Hao and Li 2014; Yoshikawa et al., 2018; Dai et al., 2019).

IGU is a new type of immunosuppressant used in RA in recent years. It can not only inhibit intracellular inflammatory factors and suppress immunity but also significantly inhibit bone resorption and bone destruction (Jiang et al., 2020; Xie et al., 2020). Joint damage is one of the important characteristics of RA, caused by abnormal bone metabolism. The balance of bone metabolism is controlled by bone resorption and bone formation, and they can be regulated by a variety of cytokines and signaling pathways. With regard to the combination of IGU and MTX, recent studies have also shown the complementarity of the two. Research by Wang et al. showed that both IGU and MTX can significantly inhibit the high expression of RANKL mRNA (*p* < 0.01), and the combination of the two drugs showed a stronger inhibitory effect (compared with MTX, *p* < 0.01; compared with IGU, *p* < 0.05). In addition, the combined drug group showed a more significant difference in inhibiting the ratio of RANKL mRNA/OPG mRNA than the single drug (*p* < 0.05) (Wang 2017). Yan and Wang (2018) also found that IGU combined with MTX can synergistically reduce the inflammatory response in patients with RA, better play the role of bone formation, and antagonize bone resorption.

This study found that the ACR20, ACR50, and ACR70 of IGU + MTX were not statistically different from that of MTX + leflunomide. However, the results of DAS28 show that the effect of IGU + MTX is better than that of MTX + leflunomide. This may be due to the fact that the MTX + leflunomide subgroup in ACR20, ACR50, and ACR70 only contains one RCT with extractable data, and the conclusion is unstable. In the future, more relevant research is needed to revise or verify this result.

### Discussion of the Source of Heterogeneity

The summary results show that the heterogeneity of ACR20 and DAS28 is high, while the heterogeneity of ACR50 and ACR70 is low. After subgroup analysis (based on the control group interventions, drug dosage and intervention duration), the heterogeneity of ACR50 and ACR70 is still low, while the heterogeneity of DAS28 and ACR20 is still high. As for the heterogeneity after subgroup analysis, we considered that the heterogeneity may be related to the following points: 1) it may be related to the patient’s baseline state of illness activity, and the baseline patient’s illness activity is not clearly stated in each study, so it cannot be further analyzed; 2) the heterogeneity of DAS28 may also be related to publication bias. In DAS28, IGU + MTX compared with IGU + adalimumab showed the opposite result from the comprehensive result. However, since only one study reported this, no definite conclusion can be drawn, and more relevant RCTs are needed to modify or verify this result. Most RCTs reported adverse reactions, but no deaths were reported. There was no difference in adverse reactions between the IGU combined with MTX and MTX alone in the blood system (leukopenia), liver function abnormalities, and gastrointestinal adverse reactions (malignant, vomiting). This shows that IGU combined with MTX will not cause additional infection or gastrointestinal adverse reactions to patients.

### Safety of IGU + MTX

Safety analysis showed that compared with MTX + tripterygium and MTX only, there was no statistically significant difference in the incidence of adverse events in IGU + MTX. Compared with MTX + leflunomide, the incidence of adverse events in IGU + MTX was lower. Current research also shows that compared with other DMARDs, IGU is safe and suitable for long-term use. For example, a multicenter, randomized, double-blind, controlled trial showed that the IGU group had a lower incidence of adverse reactions than the MTX group after 24 weeks of treatment (Lu et al., 2009). The long-term application of IGU has relatively mild adverse reactions and a low incidence. The most common adverse reactions are gastrointestinal reactions and elevated liver enzymes. Tsuneyo Mimori et al. conducted a 52-week multicenter, prospective observational, phase IV clinical study in Japan, which proved that the incidence of IGU adverse reactions reached a peak after about 4 weeks of treatment. Subsequently, the incidence of various adverse reactions did not increase over time (Mimori et al., 2019). Our previous RCT showed that during 52 weeks of treatment, IGU combined with MTX therapy is safer than traditional leflunomide combined with MTX therapy. The incidence of overall adverse events, alanine aminotransferase (ALT)/aspartate aminotransferase (AST) that reflect liver function increased, and the rate of leukopenia was lower (Tian et al., 2020). Our previous RCT showed that during 52 weeks of treatment, IGU combined with MTX therapy is safer than MTX + leflunomide therapy. The incidence of overall adverse events, the incidence of ALT/AST elevation, and leukopenia decrease are lower (Tian et al., 2020).

A recently published multicenter, prospective, real-world study of IGU in the treatment of RA also showed its better safety. The data come from the participation of 48 hospitals in China, and a total of 1,759 patients with active RA were enrolled. It showed that the incidence of ≥ grade 3 adverse events was 3.4%, and only 0.7% (13/1751) of serious adverse events were related to IGU. Compared with previous studies, no new adverse reactions were seen in this large sample study. Meanwhile, there is no obvious correlation between the patient’s age and the occurrence of adverse reactions, so it can be considered that IGU has good safety in the treatment of elderly RA patients (Mu et al., 2021). In the non-RCT of IGU combined with MTX in the treatment of RA, Okamura et al. (2015) found that the adverse reactions of this combination were mainly interstitial pneumonia and liver dysfunction (alp increase). Ishiguro et al. (2013) found that the adverse reactions were elevated liver enzymes (elevated ALS), stomatitis, pharyngitis, decreased lymphocyte count, decreased white blood cell count, and decreased red blood cell count. It can be seen that the adverse reactions of IGU combined with MTX in the treatment of RA still exist, and more clinical trial studies are needed to improve the efficacy and safety. It is necessary to expand the sample size and prolong the observation time to further evaluate its clinical efficacy and safety.

### The Strengths and Limitation of this Review

Compared with a previous review (Chen et al., 2021), the strength of this systematic review and meta-analysis is that this is the newest evaluation of the efficacy and safety of IGU + MTX for RA patients. This study also used subgroup analysis to compare the efficacy and safety of IGU + MTX versus different control groups (MTX only, MTX + leflunomide, MTX + tripterygium, MTX + adalimumab). This study also conducted an evidence quality assessment to provide clinical information on the use of IGU + MTX. This study also adopted a more rigorous risk of bias assessment and a comprehensive analysis of 31 RCTs.

The limitation of this study is that most studies have high or unclear risk of bias in random sequence generation, allocation concealment, blinding, and incomplete outcomes, which affects the accuracy of the results and the evidence’s grade. Meanwhile, most of the outcomes have high heterogeneity, and the heterogeneity does not decrease after subgroup analysis (such as DAS28). This may be related to the fact that the dosage of the medication is not exactly the same; the basic treatment, the treatment process, and the observation time are different; and the baseline data of the patients in different RCTs are different. In the future, more RCTs from different regions and nationalities with clear random sequence generation methods, allocation concealment, and blinding are needed to modify or verify the results. In addition, the country distribution of RCTs will affect the spread of evidence. The RCTs included in the meta-analysis of this study are mainly from China and Japan. The main reason is that as a new drug, IGU has recently been approved for marketing in China and Japan, and clinical researchers and patients have more convenient access to drugs. Especially in 2017, IGU entered the new version of China’s National Medical Insurance Catalogue (Category B) (Jin and Luo 2020), and its reach was further expanded, reducing the personal burden of RA patients. Since 2017, the RCT of IGU combined with MTX in the treatment of RA has increased rapidly, and it mainly comes from China, and the applicability of the evidence is mainly extrapolated to Asian countries.

### Implications for Future Research

In future clinical practice, in addition to combining MTX, RCT of IGU combined with other conventional synthetic DMARDs (csDMARDs) can also be carried out, which also shows good clinical efficacy advantages. Dai et al. found that compared with leflunomide treatment, the improvement of DAS28, joint function indexes, serum inflammation indexes, and bone metabolism indexes of combined treatment with leflunomide and IGU was more significant (*p* < 0.05) (Dai et al., 2019). A retrospective analysis by Li et al. also showed that after 12 weeks of treatment with other csDMARDs (such as sulfasalazine, hydroxychloroquine sulfate, leflunomide) combined with IGU, the DAS28, RF, CRP, ESR, and morning stiffness of RA patients with chronic interstitial pneumonia were significantly improved compared to those before treatment, and the incidence of adverse reactions was low (Hao and Li 2014). In addition, IGU can also be combined with biological disease-improving antirheumatic drugs (bDMARDs) for the treatment of patients with poor efficacy of biological agents (Yoshikawa et al., 2018). For example, in patients with poor response to tocilizumab, combined IGU treatment can significantly improve their disease activity (DAS28, CDAI, and EULAR response criteria), thereby effectively controlling the disease (Ebina et al., 2019). In summary, future studies can explore the efficacy and safety of IGU combined with various DMARDs (such as ABC) or biological agents and provide new reference information for clinical treatment.

## Conclusion

1) Compared with the MTX alone subgroup, IGU + MTX has obvious advantages in improving the compliance rate of patients with ACR20, ACR50, and ACR70. 2) In terms of secondary outcomes such as the number of tender joints, the number of swollen joints, ESR, and CRP, IGU + MTX is more effective. 3) In terms of adverse reactions, compared with the MTX alone subgroup and MTX + MTX + tripterygium, IGU + MTX does not increase the risk of infection, abnormal liver function, nausea, and vomiting in RA patients. IGU + MTX is safer than IGU + MTX + leflunomide. In the future, especially for some RA patients with poor efficacy or poor tolerance of MTX, tripterygium or leflunomide, IGU + MTX can be used as an alternative treatment. 4) When the intervention method is (IGU 25 mg Bid, MTX 10–25 mg once a week), and the intervention lasts for at least 12 weeks, the curative effect may be achieved without obvious adverse events (Shrestha et al., 2020).

## Data Availability

The original contributions presented in the study are included in the article/Supplementary Material; further inquiries can be directed to the corresponding authors.
