# Supplementary material for: The Effect and Safety of Iguratimod Combined With Methotrexate on Rheumatoid Arthritis: A Systematic Review and Meta-Analysis Based on a Randomized Controlled Trial
Source: Front Pharmacol. 2022 Jan 18;12:780154. doi: 10.3389/fphar.2021.780154 (PMC8804504; doi:10.3389/fphar.2021.780154)
Supplement: Supplementary file 2 [file Table1.DOCX]

**Table S1.** Search Strategies

| **PubMed** | (Iguratimod OR Alamode OR T-614 OR C17H14N2O6S OR CAS 123663-49-0 OR IGU OR 3-Formylamino-7-methylsulfonylamino-6-phenoxy-4H-1-benzopyran-4-one)  AND  (methotrexate derivative OR methotrexate OR methotrexate gamma aspartic acid OR methotrexate polyglutamate)  AND  (Rheumatoid arthritis OR Arthritis, Rheumatoid)  AND  (random* controlled trial [pt] OR controlled clinical trial* [pt] OR randomized [tiab] OR placebo [tiab] OR drug therapy [sh] OR random* [tiab] OR trial* [tiab] OR group* [tiab])  NOT  (animals [mh] NOT humans [mh]) |
| --- | --- |
| **EMBASE** | 1 'Iguratimod'  2 'Alamode'  3 ('T-614' or 'C17H14N2O6S' or 'IGU').ti,ab.  4 1 or 2 or 3  5 'methotrexate derivative'  6 'methotrexate'  7 'methotrexate gamma aspartic acid'  8 'methotrexate polyglutamate'  9 5 or 6 or 7 or 8  10 'Arthritis, rheumatoid'/exp  11 'Rheumatoid arthritis'  12 ('RA').ti,ab.  13 10 or 11 or 12  14 4 and 9 and 13  15 'randomized controlled trial'  16 'single blind procedure' or 'double blind procedure'  17 'crossover procedure'  18 15 or 16 or 17  19 14 and 18 |
| **Web of Science** | (Iguratimod OR Alamode OR T-614 OR C17H14N2O6S OR CAS 123663-49-0 OR IGU OR 3-Formylamino-7-methylsulfonylamino-6-phenoxy-4H-1-benzopyran-4-one)  AND  (methotrexate derivative OR methotrexate OR methotrexate gamma aspartic acid OR methotrexate polyglutamate)  AND  (Rheumatoid arthritis OR Arthritis, Rheumatoid)  AND  (randomized controlled trial [pt] OR controlled clinical trial [pt] OR trial [tiab] OR clinical trials as topic [mesh: noexp] OR Clinical Trial OR random* [tiab] OR random allocation [mh] OR single-blind method [mh] OR double-blind method [mh]) |
| **Medline** | (Iguratimod OR Alamode OR T-614 OR C17H14N2O6S OR CAS 123663-49-0 OR IGU OR 3-Formylamino-7-methylsulfonylamino-6-phenoxy-4H-1-benzopyran-4-one)  AND  (methotrexate derivative OR methotrexate OR methotrexate gamma aspartic acid OR methotrexate polyglutamate)  AND  (Rheumatoid arthritis OR Arthritis, Rheumatoid)  AND  (randomized controlled trial OR controlled clinical trial OR trial OR Clinical Trial OR random* OR random allocation OR single-blind method OR double-blind method) |
| **Clinicaltrials.gov** | Condition or disease: Rheumatoid arthritis OR Arthritis, Rheumatoid  Other terms: (Iguratimod OR Alamode OR T-614 OR C17H14N2O6S OR CAS 123663-49-0 OR IGU OR 3-Formylamino-7-methylsulfonylamino-6-phenoxy-4H-1-benzopyran-4-one) AND (methotrexate derivative OR methotrexate OR methotrexate gamma aspartic acid OR methotrexate polyglutamate) |
| **the Cochrane library** | 1 Iguratimod  2 Alamode  3 T-614 or C17H14N2O6S or IGU  4 1 or 2 or 3  5 methotrexate derivative  6 methotrexate  7 methotrexate gamma aspartic acid  8 methotrexate polyglutamate  9 5 or 6 or 7 or 8  10 Arthritis, rheumatoid/exp  11 Rheumatoid arthritis  12 RA  13 10 or 11 or 12  14 4 and 9 and 13  15 randomized controlled trial  16 single blind procedure or double blind procedure  17 crossover procedure  18 15 or 16 or 17  19 14 and 18 |
| **CNKI** | ( SU='艾拉莫德' OR SU=' T-614')  AND  ( SU='甲氨蝶呤')  AND  (SU='类风湿性关节炎') |
| **Wanfang** | (艾拉莫德 OR T-614)  AND  (甲氨蝶呤)  AND  (类风湿性关节炎) |
| **VIP** | (M=艾拉莫德OR M= T-614)  AND  (M=甲氨蝶呤)  AND  (类风湿性关节炎) |
| **CBM** | ("艾拉莫德"[常用字段:智能] OR "T-614"[常用字段:智能])  AND  ("甲氨蝶呤"[常用字段:智能])  AND  ("类风湿性关节炎"[常用字段:智能]) |
